# Calcium Homeostasis in Myogenic Differentiation Factor 1 (MyoD)-Transformed, Virally-Transduced, Skin-Derived Equine Myotubes

**DOI:** 10.1371/journal.pone.0105971

**Published:** 2014-08-22

**Authors:** Marta Fernandez-Fuente, Cesare M. Terracciano, Pilar Martin-Duque, Susan C. Brown, Georges Vassaux, Richard J. Piercy

**Affiliations:** 1 Comparative Neuromuscular Diseases Laboratory, Department of Clinical Sciences and Services, Royal Veterinary College, London, United Kingdom; 2 Laboratory of Cell Electrophysiology, Imperial College London, Myocardial Function, National Heart and Lung Institute, Hammersmith Hospital, London, United Kingdom; 3 Universidad Francisco de Vitoria, Facultad de Ciencias Biosanitarias: Pozuelo de Alarcón (Madrid), Madrid, Spain; 4 Comparative Biomedical Sciences, Royal Veterinary College, London, United Kingdom; 5 Laboratoire TIRO, UMRE 4320, iBEB, DSV, Commissariat a’ l’Energie Atomique, Nice, France; University of Minnesota Medical School, United States of America

## Abstract

Dysfunctional skeletal muscle calcium homeostasis plays a central role in the pathophysiology of several human and animal skeletal muscle disorders, in particular, genetic disorders associated with ryanodine receptor 1 (RYR1) mutations, such as malignant hyperthermia, central core disease, multiminicore disease and certain centronuclear myopathies. In addition, aberrant skeletal muscle calcium handling is believed to play a pivotal role in the highly prevalent disorder of Thoroughbred racehorses, known as Recurrent Exertional Rhabdomyolysis. Traditionally, such defects were studied in human and equine subjects by examining the contractile responses of biopsied muscle strips exposed to caffeine, a potent RYR1 agonist. However, this test is not widely available and, due to its invasive nature, is potentially less suitable for valuable animals in training or in the human paediatric setting. Furthermore, increasingly, RYR1 gene polymorphisms (of unknown pathogenicity and significance) are being identified through next generation sequencing projects. Consequently, we have investigated a less invasive test that can be used to study calcium homeostasis in cultured, skin-derived fibroblasts that are converted to the muscle lineage by viral transduction with a MyoD (myogenic differentiation 1) transgene. Similar models have been utilised to examine calcium homeostasis in human patient cells, however, to date, there has been no detailed assessment of the cells’ calcium homeostasis, and in particular, the responses to agonists and antagonists of RYR1. Here we describe experiments conducted to assess calcium handling of the cells and examine responses to treatment with dantrolene, a drug commonly used for prophylaxis of recurrent exertional rhabdomyolysis in horses and malignant hyperthermia in humans.

## Introduction

Skeletal muscle contraction involves highly specialised and tightly regulated mechanisms that convert electrochemical signals to mechanotransduction. A crucial element within this machinery is the interaction between sarcolemmal voltage-gated L-type Ca^2+^ channels (dihydropyridine receptors - DHPR) and sarcoplasmic Ca^2+^ channels - mainly ryanodine receptors (RYR1). Upon depolarization of the skeletal muscle transverse tubule (T-tubule) membrane, DHPRs interact with RYR1, activating a massive release of calcium from the sarcoplasmic reticulum (SR) into the cytoplasm. This conversion of an electrical to a chemical signal (a Ca^2+^ transient) is essential for excitation–contraction coupling (ECC): calcium released into the cytoplasm during ECC-initiation enables contraction of myofibrils via ATP hydrolysis from myosin ATPases following a calcium-dependent conformational change in the troponin-tropomyosin complex [1]–[3]. Relaxation, following myofibre contraction, occurs with calcium re-uptake by the SR via Ca^2+^ ATPase (SERCA1) pumps. Gradual depletion of SR calcium storage activates a calcium entry pathway through the plasma membrane known as store-operated calcium entry (SOCE) that can be activated during intensive exercise [4]–[6]. In addition, relatively small amounts of calcium can enter muscle cells through a prolonged voltage-dependent (excitation) -coupled calcium entry mechanism (ECCE) [7].

Several human and animal myopathies are associated with dysfunction of skeletal muscle calcium regulation. These include malignant hyperthermia (MH), a life-threatening form of rhabdomyolysis associated with use of volatile anaesthetic and certain neuromuscular blocking agents that occurs in humans [8], [9], dogs [10], [11] and horses [12], [13], and in porcine stress syndrome, a disorder that leads to major losses to the pork industry due to its association with poor quality (pale, soft, exudative) meat [14], [15]. Both MH and porcine stress syndrome are associated with RYR1 mutations, as are the human muscle disorders, central core disease (CCD), multiminicore disease (MmD), certain nemaline rod-associated myopathies and some centronuclear myopathies [16]–[24]. In CCD, evidence suggests 2 possible pathophysiological mechanisms: RYR1-associated calcium leakage [25] and uncoupling of DHPR-RYR1 [23], [26], [27]. Brody disease (a myopathy associated with recessive mutations in SERCA1 and delayed muscle relaxation) is reported in humans and cattle [28]–[30]. In addition, some human patients are reported with phenotypes suggestive of calcium handling defects, including MH, but without detectable mutations in RYR1 or other related genes [31]. Similarly, an inherited and apparent calcium handling defect that affects 5–7% of Thoroughbred racehorses worldwide, known as recurrent exertional rhabdomyolysis (RER) [32]–[34], shares pathophysiological features with MH, but mutations in RYR1, SERCA1 and DHPR have been excluded [35]. A likely-identical disorder also occurs in Standardbred racehorses with a similar prevalence [36]. RER in horses is widely treated prophylactically with orally-administered dantrolene, an RYR1-antagonist that at high doses can induce transient paresis [37] but that reduces severity of exercise-induced muscle damage in some affected horses [38]. Dantrolene is also used to treat episodes of MH in humans, conceivably by reducing voltage dependent (excitation) coupled calcium entry [7], [39] but the mechanism of action in each species is poorly understood.

The RYR1 cDNA’s large size (∼15 kb) traditionally made mutational analysis (and consequent study of the downstream effect of defects in the gene), extremely labour intensive. With the advent of next generation sequencing technologies it is feasible rapidly to sequence the entire gene; however, and consequently, many single nucleotide polymorphisms (SNPs) have been found in RYR1 and without some functional assessment, it is sometimes difficult to determine their pathogenicity, if any [40], [41].

Functional assessment of MH in humans and RER in horses traditionally relied on *in* *vitro* contraction testing (IVCT) of isolated strips of skeletal muscle when exposed to caffeine and other agents [42]. Furthermore, diagnosis in other calcium-related human disorders, such as CCD and MmD still relies heavily on muscle biopsy [43]. However, muscle biopsy in general, and the IVCT (which requires an open biopsy) in particular, are relatively invasive. Consequently, clinicians and researchers have looked for alternative cell culture systems that can model calcium handling *in* *vitro*, with a view to gaining insight into disease pathophysiology, accurate diagnosis and for investigation of novel treatments. This has been achieved in cultured myoblasts (derived from muscle biopsy specimens) [44], [45], but a system that instead utilises fibroblasts derived from small skin biopsy samples [46], [47], is attractive, because the sampling method is less invasive, and the primary cells are more robust in culture than myoblasts. Several groups, including our own have converted skin derived fibroblast-like cells to muscle cells (myotubes) in culture, utilising viral transduction with muscle-specific transcription factors [48], [49]. For example, calcium handling - specifically caffeine and chloro-m-cresol responses - has been studied in human skin-derived fibroblasts-converted to myotubes through forced (adenovirally-mediated) expression of MyoD [46], [50]–[53]. The method is especially attractive in the paediatric setting and in animals where the more invasive muscle biopsy, might affect value or influence athletic training.

Despite previously published work, there has been very little functional analysis of skin-derived myotubes in relation to RYR1-associated physiology; therefore, an in-depth analysis of their excitation-calcium release dynamics seems essential if they are to be used in confirming or characterising the phenotype of patients in whom RYR1 mutations or a defect in calcium handling is suspected. Validation of the use of virally-transduced, skin-derived fibroblasts in assessment of human and animal myopathies is extremely important, since this *in* *vitro* approach has great potential in both clinical diagnostic and research settings. In our earlier work, we reported a lentivirus-based system that converted cultured equine skin-derived fibroblasts to myotubes through expression of equine MyoD [48]. More recently, we reported the generation of an adenoviral vector expressing (equine) MyoD that also transforms both equine and human skin-derived fibroblasts to myotubes [49]. Here we describe a comprehensive characterisation of this system, in the context of the modelling of skeletal muscle calcium homeostasis in horses.

## Materials and Methods

### Animals and cell culture

Primary equine fibroblast-like cells were derived from skin biopsy samples collected under local anaesthesia with sterile 4 mm disposable punch instruments, from the left side of the mid neck crest in 2, two-year-old, Thoroughbred racehorse geldings. Muscle biopsy samples were obtained from 2 three-year-old Thoroughbred geldings, euthanased for reasons unrelated to this study. The procedure was approved by the Royal Veterinary College local Ethics’ committee and was performed with adherence to a Home Office Project license (PPL 70/6523) according to the Animal (Scientific Procedures) Act 1986 of the United Kingdom. Horses were selected on the basis of their being normal on physical examination and through their never having been observed to have a clinically-detectable episode of RER during their racing career; subclinical RER was considered highly unlikely on the basis of plasma creatine kinase and aspartate amino transferase activities remaining within normal laboratory limits when tested approximately fortnightly throughout an entire racing season (data not shown).

Skin samples were collected into sterile tissue culture growth medium (see below) and transported to the laboratory and maintained at 4°C. Within 24 hours, biopsy specimens were cut into 1-mm cubes with a sterile scalpel blade and placed into the centre of 3.5 cm tissue culture dishes containing culture medium and incubated at 37°C in an incubator containing 5% CO_2_. Skin and muscle samples were routinely cultured in normal growth medium consisting of Dulbecco modified Eagle medium, 10% foetal bovine serum (FBS; PAA), penicillin-streptomycin (1 U/mL and 1 µg/mL, respectively; Life Technologies) and 2 mM L-glutamine (Life Technologies). Within 5–7 days, spindle-shaped cells were readily visible migrating outward from the minced tissue. Cells were passaged routinely (fewer than 10 times) until cell numbers were high enough for subsequent experiments.

Equine cells (passage<5) and human embryonic kidney cell line (293T) cells were routinely cultured in normal growth medium (as above). 20% FBS-containing media was used for primary myoblast culture (as derived and described previously [49]) prior to their differentiation.

### Virus production and myogenic conversion

Adenovirus particles expressing bicistronic eqMyoD cDNA and eGFP from the same CMV promoter, were produced using the system developed by the Vogelstein lab [54] as we previously described [49]. Viral titre was determined (through infection of 293T cells) to be 2.4×10^9^ infectious units/ml; particles were stored in aliquots at −80**°**C until use.

Matrigel (1∶1000, Becton-Dickinson)–coated, glass-bottomed 3.5 cm diameter dishes (MatTek Corporation) were seeded with 5×10^4^ equine skin fibroblast–like cells/well and transduced with the adenovirus (multiplicity of infection (MOI) of 5) [49] in a final volume (50 µL drop) on the central glass coverslip for 4 hours after which virus-containing medium was replaced by 2 ml fresh growth medium. 48 hours after infection, medium was replaced with differentiation medium (MegaCell; Sigma), 2% FBS, penicillin-streptomycin (1 U/ml and 1 µg/ml, respectively), L-glutamine (2 mM; Sigma), 1x Non-essential Amino acids (Sigma) and beta mercaptoethanol (0.7 µl/100 ml, Sigma). Approximately 1 week after infection, the cells’ morphological characteristics had changed, becoming more elongated with the formation of myotubes. 14–21 days later, cells were used for assessment of calcium homeostasis by fluorescence.

### Calcium measurements

Myotubes (either 14 or 21 days following transduction), were loaded with Indo-1 AM (Molecular Probes) at a concentration of 10 µM for 20 min at 37°C and then incubated for at least 1 h in normal growth medium in order to ensure complete intracellular de-esterification of Indo1-AM. Cells were subsequently analyzed while being superfused with normal Tyrode solution (140 nM NaCl, 6 mM KCl, 10 mM glucose, 10 mM HEPES, 1 mM Mg_2_Cl and 2 mM CaCl_2_; pH 7.4) and at a constant temperature of 37°C. After 5 min accommodation, cells were excited at 360 nm using a 100 W xenon lamp (Nikon); cellular fluorescence signals were recorded simultaneously at 405 nm (F405) and 485 nm (F485) using photomultiplier tubes (PMT, PTI814 Thorn EMI). Background fluorescence subtraction was routinely performed using a cell-free region as zero fluorescence. Changes in cytoplasmic calcium concentration were expressed as changes in the ratio (R = F405/F485) thereby avoiding the influence of photo-bleaching.

Cells were exposed to calcium-modifying agents through use of a solenoid switcher device. At least 5 myotubes were studied per dish. Maximal amplitudes of caffeine peaks after caffeine stimulation were measured using Clampfit software (Axon Instruments). Between 20 and 40 myotubes in total were studied for caffeine exposure experiments, from 4 different dishes. Thapsigargin (SERCA blocker) experiments (n = 16 tubes from two dishes) were conducted with an initial stimulation via a pulse of 20 mM caffeine; after Ca^2+^ concentration returned to baseline, the superfusate was switched to normal Tyrode or to Ca^2+^-free Tyrode solution, and then to 300 nM thapsigargin (in the corresponding buffer, Ca^2+^-free or normal Tyrode solution) for 3 minutes, after which two further 20 mM caffeine stimulations were recorded. Similar protocols were used when using tetracaine (n = 10 tubes from two dishes) and dantrolene (n = 15 tubes from two dishes) (50 µM and 10 µM respectively) to modify caffeine responses. To confirm that dantrolene had no direct effect on Indo-1 fluorescence, we compared the fluorescence emitted by Indo-1 pentapotassium salt, at a range of calcium concentrations (0 to 9 mM) in the presence and absence of dantrolene (10 µM).

### Statistical analysis

Caffeine-response data at 2 and 3 weeks are expressed as mean ± S.E.M. Differences in data among groups were examined by 2 way repeated measurement ANOVA followed by Tukey’s test for post hoc analysis. Differences were considered statistically significant when p<0.05.

## Results

### Caffeine responses in equine myotubes

**Figure 1:**
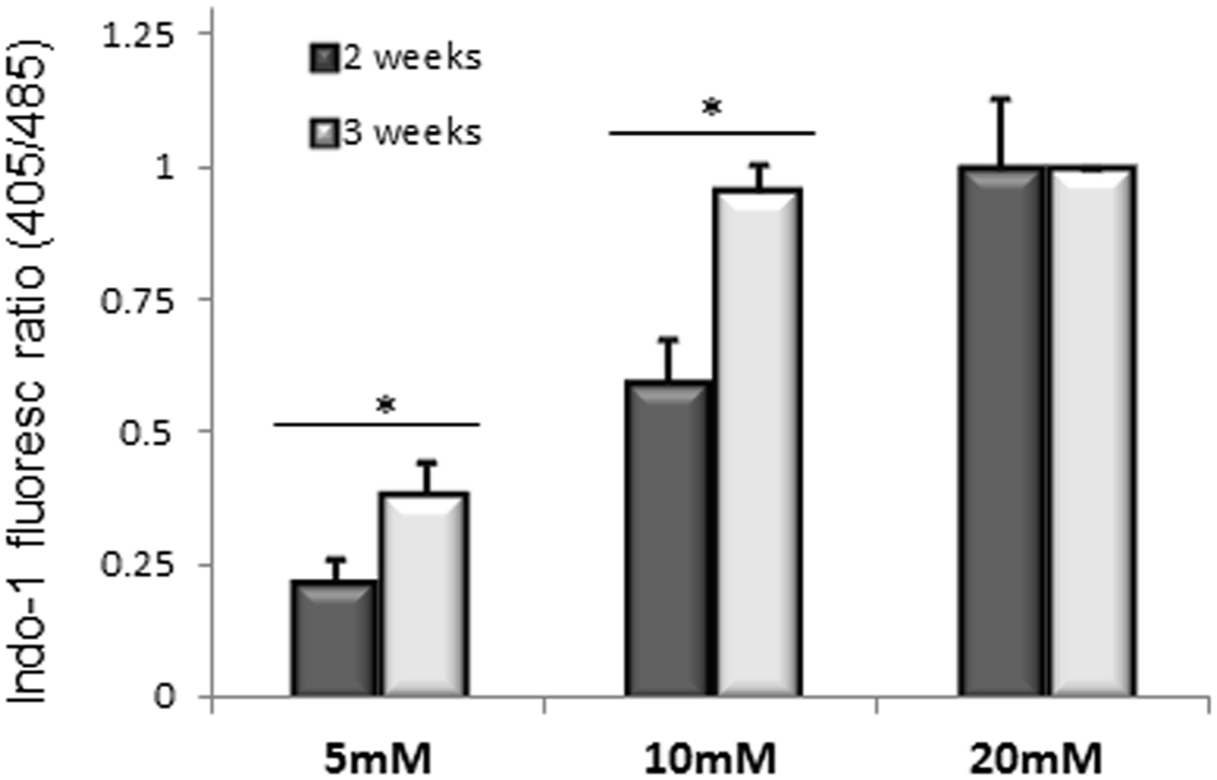
Caffeine dose response. Histogram of Indo-1 response to increasing concentrations of caffeine in adenovirally-transduced equine myotubes. Results show mean (+/−1 SEM) derived from 40 myotubes from 2 control horses sequentially exposed to 5, 10 and 20 mM caffeine at 2 and 3 weeks’ differentiation.

**Figure 2:**
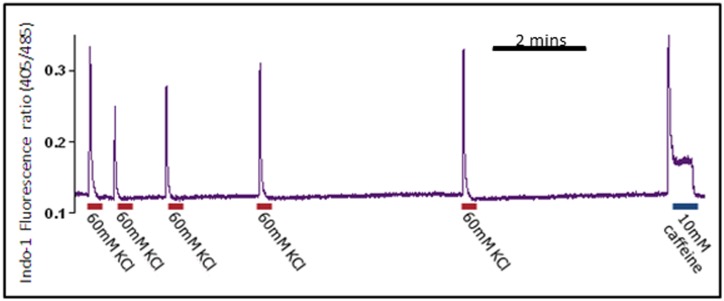
Depolarisation with KCl. Representative experiment where skin-derived equine myotubes were subjected repeatedly to depolarization with 60 mM KCl (red line). 10 mM caffeine responses (blue line) are shown for comparison. Note that responses are reproducible and near maximal within approximately 4 minutes.

To assess if calcium homeostasis of skin-derived myotubes is affected by differentiation time, we studied the response of the equine cultures to caffeine, after 2 or 3 weeks of differentiation. Figure 1 shows peak calcium release responses when cells were exposed to 5, 10 and 20 mM caffeine at each of the 2 time points. We observed significantly greater calcium release (P<0.0001) at 3 weeks compared to 2 weeks at two intermediate caffeine concentrations (5 and 10 mM) (Figure 1). As expected [49], we observed a dose-response in caffeine-induced calcium release (Figure 1), with a plateau (in 3 week differentiated cells) at around 10 mM caffeine. By 3 weeks of differentiation, caffeine responses were robust and reproducible: a functional excitation-calcium release-mechanism was indicated by the near maximal (in comparison with 20 mM caffeine) transients when cells were depolarised with 60 mM KCl (Figure 2). Within any one myotube, near maximal recovery to the KCl-induced response occurred after approximately 4 minutes (Figure 2). Cells remained responsive to sequential KCl-induced depolarisation or caffeine-induced calcium release as long as 30 minutes (when experiments were terminated); there was no apparent reduction in the amplitude of responses during this time (not shown).

### Studies using calcium channel agonists/antagonists

**Figure 3:**
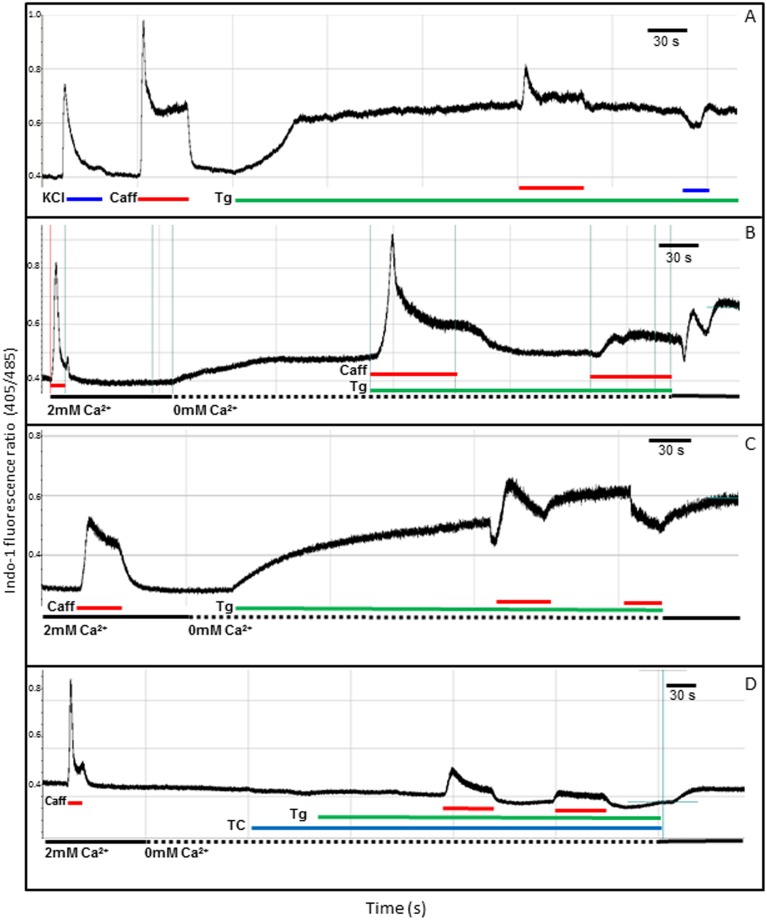
Calcium responses of skin-derived myotubes, treated with specific agonists and antagonists. A) Typical transients recorded following depolarisation with 60 mM KCl (blue line) and after exposure to 20 mM caffeine (red lines). The responses are almost abolished when the sarcoplasmic reticulum ATP-ase (SERCA-1) is blocked with thapsigargin (green line). Note that thapsigargin treatment induces an increase in Indo-1 fluorescence, that reveals leakage of SR calcium into the cytoplasm when SERCA-1 activity is blocked. B) Effect of thapsigargin on the response of skin-derived myotubes to caffeine, using calcium-free solution (dashed line). The increase in Ca^2+^ signal after the blockage of SERCA-1 supports the hypothesis of a leaky RYR-1 channel. C) Thapsigargin treatment was assayed on myotubes derived from primary, differentiated myoblasts, confirming that a similar leakage from the SR also occurs in these cells. D) When tetracaine is added to the perfusion system prior to the initiation of thapsigargin, the leakage is completely blocked, suggesting that RYR-1 is responsible for the leakage of SR calcium.

**Figure 4:**
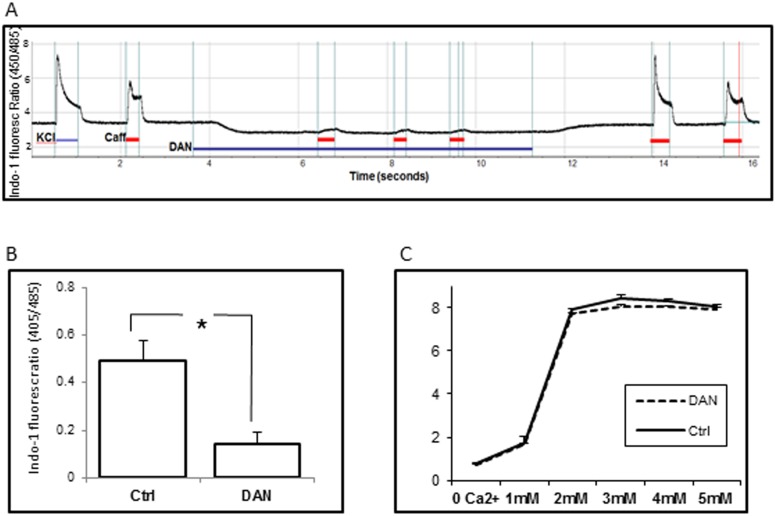
Effect of dantrolene on calcium transients. A) Depolarization with 60 mM KCl and 20 mM caffeine in normal buffer, followed by exposure to sequential exposures to caffeine in the presence of 10 µM dantrolene. B) The amplitude of the caffeine transient in the absence or presence of dantrolene. Note that dantrolene almost completely inhibits the ability of the myotubes to respond to caffeine (p<0.05) and lowers the cytoplasmic calcium concentration of resting myotubes. C) Indo-1 fluorescence at increasing calcium concentration, in the presence or absence of 10 µM dantrolene. Note that there was no direct effect of dantrolene on Indo-1 fluorescence.

To further characterize calcium homeostasis in our equine skin-derived cells, we used various agonists and antagonists of calcium regulatory proteins in calcium-containing and calcium-free solutions (Figures 3 and 4). Thapsigargin, a non-competitive antagonist of SERCA1 [55], almost completely abolished caffeine responses (Figure 3A). During treatment with thapsigargin there was an increase in Indo-1 basal fluorescence, revealing accumulation of cytoplasmic calcium when SERCA-1 activity is blocked. To ascertain whether this increase in calcium concentration was associated with sarcoplasmic Ca^2+^ leakage or to an influx of extracellular Ca^2+^, we repeated the experiments using calcium-free bathing solutions. In absence of extracellular calcium, thapsigargin had the same effect: it induced a clear increase in Indo-1 fluorescence, indicative of a release of calcium from intracellular stores in skin-derived myotubes (Figure 3B). In order to determine whether this calcium leak was unique to skin-derived myotubes, we performed the same experiments in 15 day-old, primary equine myotubes derived from equine primary skeletal muscle cultures: our experiments revealed that these cells also had detectable SR leakage of calcium when SERCA was blocked (Figure 3C). Finally, we investigated whether this calcium leak occurred via RYR receptors: leakage through RYR can be blocked with tetracaine, an agent that inhibits spontaneous calcium release at low concentration [56]. In our model, tetracaine completely blocked the leak of sarcoplasmic calcium (Figure 3D), suggesting that calcium leakage occurs by way of the RYR receptor, and consistent with results observed in skeletal and cardiac muscle, which express RYR-1 and RYR-2 respectively [56], [57].

As expected, dantrolene diminished the amplitude of the caffeine-induced calcium transient in skin-derived myotubes (Figures 4 A and B). We also detected a reduction in Indo1 fluorescence ratio (and hence cytoplasmic calcium concentration) in the presence of dantrolene (Figure 4): in order to confirm that this reduction was not an artefact associated with a direct effect of dantrolene on the Indo1 fluorescence ratio, we examined the influence of 10 µM dantrolene on the Indo1 fluorescence ratio at increasing predetermined calcium concentrations (0–9 mM): dantrolene had no effect on the Indo1 fluorescence signal (Figure 4C) confirming that dantrolene induced a reversible reduction in basal cytoplasmic calcium concentration in dermally-derived equine myotubes.

## Discussion

There are several human and animal myopathies that involve disturbances in the regulation of skeletal muscle calcium homeostasis and hypersensitivity to agents that influence RYR1 channel opening, in particular, caffeine and halothane [16]–[18]. The precise nature of the perturbation can differ, even in patients with mutations in the same gene. For example, in some CCD-affected human patients, RYR1 mutations appear to induce an uncoupling between DHPR and RYR1, while in other patients with MH, the mutation makes the RYR1 channel leaky, leading to raised cytosolic calcium concentrations [58], [59]. For many suspected RYR1 mutations however, the precise influence on muscle calcium homeostasis is unclear: when diagnosis largely depended on histological diagnosis following muscle biopsy, the IVCT was considered the gold standard test, but with the advent of direct next generation sequencing in patient diagnosis, there is likely reluctance to perform this more invasive procedure. In the human paediatric setting, or in valuable horses in full athletic training, use of muscle biopsy poses an additional diagnostic dilemma. A minimally-invasive method that can be used to investigate calcium homeostasis defects associated with known, or suspected myopathies is therefore especially attractive. Previously we revealed that an adenovirus expressing MyoD can convert both human and equine skin-derived fibroblasts to fusion competent myotubes, and further, that the cells respond similarly to primary myoblasts when exposed to incremental caffeine concentrations [49]. However, in order to understand the cellular model’s potential for accurately defining defects in DHPR-RYR1 coupling, and in particular, RYR calcium leakage, it was important to evaluate in detail these aspects of the cells’ physiology. The model’s validation seems especially important given that similar techniques have been used previously to model calcium homeostasis defects in human patients [23].

In the current experiments, we conducted a detailed characterisation of calcium handling in dermally-derived myotubes using a range of agonists/antagonists of the main channels involved in EC-coupling. We detected the classical calcium release pathway mediated by RYR activation following cellular depolarisation and therefore revealed a functional coupling between DHPR and RYR proteins; however, in addition, our data supports the existence of a second, less well-defined and passive SR calcium efflux pathway, previously referred to as RYR calcium leak, which might occur through various proteins including RYR and translocons [58] [60], [61]. We demonstrated these skin-derived myotubes’ leak, by blocking SERCA-1 with thapsigargin and further, by showing that the rise in intracellular calcium occurred, even when the cells were maintained in calcium free buffer. We also revealed that the same response is seen in myotubes derived from primary myoblasts. Finally, we confirmed that this passive efflux of calcium was blocked by tetracaine, an RYR1 antagonist [60], [61], revealing that the leak occurred via this receptor and was SR-derived [58], [62], [63].

The on-going recycling of calcium between SR and the cytoplasm mediated by this leak is overwhelmed by the massive release of SR that occurs during cell depolarisation or when cells are stimulated by caffeine. Interestingly, and as suggested previously [64] the extent of the leak appears to contribute to the basal cytoplasmic calcium concentration, since calcium concentration reduced when cells (in the resting state) were exposed to the RYR1 antagonist, dantrolene, thereby excluding excitation-coupled calcium entry [7], [39]. In horses, dantrolene has been used both to treat [38] and prevent RER [65], [66]. At higher doses, dantrolene can induce paresis in horses [37] presumably by limiting the extent of calcium release from the SR required for excitation-contraction coupling and as suggested by the diminished SR response to caffeine that we observed. However, our data suggest that the efficacy of dantrolene in equine RER and in MH, might relate to its lowering cytoplasmic calcium concentration in the cells’ resting state (high cell calcium concentrations can stimulate cellular proteases and lead to rhabdomyolysis [67], [68]. Although further work is required, our data suggest that the drug might play a role in the treatment or management of additional RYR1-associated human or animal myopathies or other muscle disorders with primary or secondary defects in calcium homeostasis.

To date, there had been no “in depth” studies detailing the calcium handling of dermally-derived, virally-transduced cells. Our previous [48], [49] and current work, reveals that these cells respond similarly to myotubes derived from primary muscle cultures. Whilst recognising the underlying cycling of calcium and the extent of SR calcium leakage that occurs in such cells, the system appears to offer a convenient, minimally-invasive model with which to test calcium homeostasis in patients that precludes any need for muscle biopsy. As such, the system might be more appropriate for evaluation of racehorses in training than intercostal muscle biopsy [69]; future work should be directed at comparison of responses detected in dermally-derived cells from horses and humans with known or suspected defects in cellular calcium homeostasis and controls.
